# Psychological outcomes and changes in health-related quality of life in young and middle-aged patients with first-onset stroke after rehabilitation training

**DOI:** 10.3389/fpsyt.2025.1714210

**Published:** 2026-01-05

**Authors:** Chao Han, Dahai Yu, Xueli Sun, Ziyan Liang

**Affiliations:** Department of Rehabilitation Medicine, Zibo First Hospital, Zibo, China

**Keywords:** depression, first-onset stroke, health-related quality of life, psychological outcomes, rehabilitation

## Abstract

**Objectives:**

The primary objective of this study is to determine the changes in psychological well-being and health-related quality of life (HRQOL) among young and middle-aged individuals who have experienced their first stroke and undergone structured rehabilitation training.

**Methods:**

This prospective analysis included 203 young and middle-aged individuals who had experienced their first stroke and took part in a comprehensive rehabilitation program conducted in China. Patients were classified into “Unremarkable” or “Impaired” groups based on their pre-rehabilitation scores in each scale of the SF-36, SCL-90, and BDI-II scales using established cut-off values from prior research. The rehabilitation program consisted of physical therapy, occupational therapy, and psychological counseling. The rehabilitation program began at least 4 weeks after stroke onset and was carried out over a period of 6 to 8 weeks, with participants attending five sessions per week. Neuropsychological assessments, including the SF-36, SCL-90, and BDI-II scales, were conducted before and after rehabilitation to measure changes in HRQOL, psychological outcomes, and depressive symptoms.

**Results:**

In terms of HRQOL, notable improvements were observed, particularly in the “impaired” group. Following rehabilitation, key indicators, including physical function, overall health, bodily pain, and vitality, showed considerable enhancement (P < 0.001). Regarding psychological outcomes, the “impaired” group demonstrated substantial reductions in symptoms such as aggression, anxiety, obsessive-compulsive behaviors, and paranoid ideation (P < 0.001). Furthermore, assessments using the BDI-II and SCL-90 depression scales indicated a significant decrease in depressive symptoms within the “impaired” group (P < 0.001), whereas no meaningful changes were noted in the “no significant abnormalities” group. These results demonstrate the effectiveness of rehabilitation in improving both psychological well-being and HRQOL in patients with more severe pre-rehabilitation impairments.

**Conclusion:**

Rehabilitation training significantly improves psychological outcomes and HRQOL in young and middle-aged individuals with first-onset stroke, particularly those with more severe baseline impairments. These findings support the integration of multidisciplinary rehabilitation programs in the management of stroke recovery, with significant benefits for both physical and psychological recovery.

## 1 Background

Stroke is a significant contributor to long-term disability and mortality across the globe, affecting both individuals and healthcare systems (1). It is the second leading cause of death worldwide, representing about 11.6% of all deaths in 2019 (2). Additionally, stroke is a major cause of disability, with an estimated 80 million people around the world living with its long-term consequences (3). While stroke traditionally has been considered a disease primarily affecting older individuals, recent epidemiological trends have highlighted an increasing incidence among younger populations (4, 5). Recent research suggests that strokes affect approximately 10% to 15% of individuals within the age range of 18 to 50 years (4, 5). This younger age group faces unique challenges, including long-term disability, reduced productivity, and difficulties with social and family reintegration. In China, stroke rates among young and middle-aged adults have been steadily rising, becoming an increasing cause of disability and death (6). This trend is attributed to factors such as the growing prevalence of hypertension, diabetes, smoking, and sedentary lifestyles (7, 8). An analysis of ischemic stroke in young adults (20-49) in China from 1990 to 2019 revealed a decrease in mortality and DALYs, but an increase in burden from risk factors like high body mass index, red meat intake, and air pollution (9). Males showed a faster rise in disease burden compared to females (9). This underscores the need for focused stroke prevention and rehabilitation strategies tailored to the unique challenges faced by younger individuals.

Despite advancements in acute stroke treatment and survival rates, many stroke survivors experience enduring physical, psychological, and cognitive impairments that significantly affect their quality of life (10). While much of the focus in stroke rehabilitation has been on physical recovery, mental health and emotional well-being are increasingly recognized as critical components of recovery. Psychological conditions such as depression, anxiety, and post-stroke stress are prevalent among stroke survivors, with studies showing that up to 30-50% of stroke patients experience depression (11, 12). Depression, in particular, is associated with worse functional recovery, increased disability, and poorer long-term outcomes (13). Additionally, cognitive impairments, emotional dysregulation, and reduced social engagement further compound the challenges faced by stroke survivors, making it essential to consider psychological recovery as an integral part of rehabilitation.

Health-related quality of life (HRQOL) acts as a key multidimensional indicator, reflecting various aspects such as physical health, psychological well-being, and social functioning (14). Although numerous studies have focused on physical rehabilitation post-stroke, fewer have addressed the full spectrum of HRQOL, particularly in terms of psychological outcomes. Stroke survivors frequently encounter challenges such as decreased life satisfaction, social withdrawal, and difficulties with emotional regulation, which may contribute to a further decline in their overall quality of life (15, 16). In younger individuals, these challenges may be even more pronounced due to their unique roles in family, employment, and social life. Addressing these issues through structured rehabilitation programs is critical to improving both psychological well-being and functional recovery. There is an increasing awareness of the important role that comprehensive rehabilitation programs may have in supporting both physical and psychological recovery (17). However, most studies have focused on older stroke survivors or have provided limited insights into how rehabilitation programs impact psychological outcomes and HRQOL across different age groups (18–20). To date, research on the psychological recovery of young and middle-aged stroke survivors, particularly in the context of rehabilitation, remains limited. This study seeks to contribute to the existing literature by examining the effects of structured rehabilitation programs on psychological outcomes and HRQOL in young and middle-aged individuals following their first stroke. To evaluate changes in psychological well-being and overall life quality, standardized neuropsychological tools, such as the SF-36, SCL-90, and BDI-II scales, were employed.

## 2 Methods

### 2.1 Participants enrollment

This prospective study included young and middle-aged individuals with first-onset stroke who underwent structured rehabilitation at Department of Rehabilitation Medicine, Zibo First Hospital between February 2022 and March 2025. The inclusion criteria were: (1) age ranging from 18 to 65 years; (2) first-onset stroke diagnosed by clinical and neuroimaging findings; (3) at least 4 weeks post-stroke; (4) no prior psychiatric or neurological disorders; (5) ability to provide informed consent and engage in rehabilitation. Exclusion criteria: (1) severe cognitive impairment, assessed using the Mini-Mental State Examination (MMSE) with a cutoff score of 24, which resulted in the exclusion of participants with significant cognitive deficits; (2) Physical disabilities hindering rehabilitation participation; (3) active psychiatric disorders requiring intervention; (3) concurrent rehabilitative or psychotherapeutic treatments. A total of 203 patients were enrolled and underwent comprehensive rehabilitation. Post-rehabilitation assessments were performed to evaluate changes in HRQOL, psychological outcomes, and depressive symptoms. The study was approved by the institutional review board. Informed consent was signed by all participants.

### 2.2 Procedures of comprehensive rehabilitation

All participants took part in a comprehensive and personalized rehabilitation program designed to support both physical and psychological recovery. This program encompassed three primary components: physical therapy, occupational therapy, and psychological counseling. (1) Physical therapy was conducted by licensed physical therapists and aimed to improve motor skills, strength, and coordination. The training included exercises to increase muscle strength, enhance joint flexibility, improve balance, and support gait retraining, with a focus on restoring functional movement. Each patient’s treatment plan was customized to meet their specific physical condition and rehabilitation requirements, typically consisting of five sessions per week over a period of 6 to 8 weeks. Each session lasted approximately one hour. (2) Occupational therapy was led by certified occupational therapists and focused on helping patients improve their ability to perform essential daily activities, such as dressing, eating, bathing, and grooming. Therapists used adaptive techniques and tools, such as specialized utensils, dressing aids, and mobility aids, to assist patients in regaining independence in these tasks. Therapists tailored the therapy to each patient’s specific functional impairments and provided individualized strategies for improving daily living skills. Occupational therapy sessions were conducted five times per week, with each session lasting approximately one hour. This frequency and duration allowed for consistent progress and the effective implementation of the therapeutic techniques. (3) Psychological counseling was provided by licensed clinical psychologists and focused on managing the mental and emotional challenges associated with stroke recovery. This component involved both individual and group therapy, with sessions tailored to the specific needs of each participant. Cognitive behavioral techniques (CBT), including cognitive restructuring, behavioral activation, relaxation training, and problem-solving strategies, were employed to address issues such as anxiety, depression, and post-stroke stress. For individual counseling, participants were scheduled for one-on-one sessions with licensed clinical psychologists. Group counseling sessions, involving multiple participants with similar psychological needs, were also offered to foster peer support and shared coping strategies. Psychoeducation, which was conducted in person, was integrated into both individual and group sessions. These approaches ensured that all participants received appropriate psychological support throughout their rehabilitation process. Sessions were structured to enhance coping strategies, improve emotional well-being, and provide long-term tools for managing psychological difficulties. Each session typically lasted one hour, and the structure included psychoeducation, guided practice, and real-life application of techniques. Counseling, including psychoeducation, was provided five times per week throughout the rehabilitation program. Rehabilitation training was carried out by a team of professionals, including physical therapists, occupational therapists, and clinical psychologists. Treatment plans were periodically modified in accordance with each patient’s rehabilitation progress.

### 2.3 Neuropsychological assessments and clinical data collection

Trained neuropsychologists evaluated the psychological health, depressive symptoms, and HRQOL of the participants. Psychological outcomes were assessed using the SCL-90, with depressive symptoms measured through the BDI-II. HRQOL was examined using the SF-36) The criteria for functional impairment were established based on prior research findings (21, 22). The SF-36 is a Likert scale with 36 items assessing eight domains, including physical and emotional health. It has a reliability range of 0.70 to 0.90 (Cronbach’s alpha) as previously reported (23) and was translated into Chinese using standard back-translation procedures. Results were considered abnormal if the z-score was less than −1 SD. The SCL-90, with 90 items, measures nine psychological symptoms and has a reliability range of 0.85 to 0.95. It was also translated using a rigorous back-translation process, and abnormal results were defined by a t-score > 60, with a t-score > 63 on the Global Severity Index indicating significant distress. The BDI-II is a 21-item Likert scale assessing depressive symptoms, with a reliability coefficient of 0.90. The cut-off scores for depression severity were: mild depression (≥ 14), moderate depression (≥ 20), and major depression (≥ 29). All tools were used with permission from the original developers, and translations were reviewed for cultural and linguistic accuracy. These assessments were performed both pre- and post-rehabilitation to evaluate changes in psychological functioning and quality of life. Pre-rehabilitation measurements served to establish baseline psychological states, while post-rehabilitation evaluations assessed the effectiveness of the rehabilitation program. The post-rehabilitation assessments were conducted within one week of completing the program to capture the immediate effects and assess short-term benefits before external factors could influence the outcomes. In addition to these neuropsychological tools, demographic information, medical history, and stroke severity were considered to account for confounding variables in the analysis.

Participants were classified into “impaired” and “unremarkable” groups based on their post-rehabilitation scores from the SF-36, SCL-90, and BDI-II, compared to established cut-off values. Those with scores below the cut-offs were categorized as “impaired”, while those with scores meeting or exceeding the cut-offs were classified as “non-impaired”, reflecting better rehabilitation outcomes. This classification allowed for a clear distinction between participants based on their rehabilitation progress.

### 2.4 Statistical analysis

Data analysis was conducted using SPSS version 26.0 (IBM Corp., Armonk, NY). Descriptive statistics were used to summarize baseline characteristics, with categorical variables expressed as frequencies and percentages, and continuous variables reported as means with standard deviations (SD). To compare SF-36, SCL-90, and BDI-II scores before and after rehabilitation, paired t-tests were used for normally distributed data, and the Wilcoxon signed-rank test was applied for data that did not follow a normal distribution, as determined by normality tests such as the Shapiro-Wilk test. Effect sizes were also calculated to assess the magnitude of the changes. A p-value of less than 0.05 was considered statistically significant for all analyses.

## 3 Results

### 3.1 Baseline characteristics

The baseline characteristics of the study cohort revealed that 64.53% of the participants were male, with a mean age of 50.54 years and a SD of 14.15. The mean BMI was 25.13 kg/m² (SD = 1.52), with 26.1% categorized as overweight, and 23.7% as obese. In terms of educational attainment, 26.1% of patients had no formal education, 23.7% had completed primary school, and 50.3% had received education at or above the middle school level. Employment status indicated that 77.3% of patients were employed, while 22.7% were unemployed. Smoking history was prevalent, with 32.0% being current smokers, 29.1% former smokers, and 38.9% never having smoked. Approximately 17.2% of the patients reported consuming alcohol regularly, 35.0% consumed it occasionally, and 47.8% did not drink alcohol at all. Regarding physical activity, 58.1% of patients reported sedentary behavior, while 41.9% were physically active. Comorbidities included hypertension (68.97%), diabetes mellitus (33.0%), hyperlipidemia (38.4%), and coronary artery disease (23.2%). Detailed information regarding these characteristics is presented in **Table 1**.

**Table 1:** Baseline characteristics.

Characteristics	Total (n=203)
Age (Years)	50.54 ± 14.15
Gender [n(%)]	
Male	131 (64.53)
Female	72 (35.47)
BMI (kg/m2)	25.13 ± 1.52
Education level [n(%)]	
No formal school	53 (26.10)
Primary school	48 (23.65)
Middle school or above	102 (50.25)
Employment [n(%)]	
Employed	157 (77.34)
Unemployed	46 (22.66)
Smoking history [n(%)]	
Current	65 (32.02)
Former	59 (29.06)
Never	79 (38.91)
Alcohol history [n(%)]	
Regular	35 (17.24)
Occasional	71 (34.98)
None	97 (47.78)
Exercise habits [n(%)]	
Sedentary	118 (58.13)
Active	85 (41.87)
Comorbidities	
DM	67 (33.00)
HTN	140 (68.97)
Hyperlipidemia	78 (38.42)
CAD	47 (23.15)

BMI, Body Mass Index; DM, Diabetes Mellitus; HTN, Hypertension; CAD, Coronary Artery Disease.

### 3.2 Post-rehabilitation changes in HRQOL

After rehabilitation, young and middle-aged individuals with first-onset stroke showed noticeable improvements in HRQOL across several domains of the SF-36 scale. Among those initially classified as impaired, 31.0% experienced meaningful gains in physical function, with scores improving from -3.12 ± 0.40 to -1.18 ± 0.25 (P < 0.001). Furthermore, 49.3% of patients reported significant improvements in overall health, with scores increasing from -2.96 ± 0.47 to -1.06 ± 0.79 (P < 0.001). In the bodily pain domain, 26.1% of patients showed substantial improvement, with scores rising from -2.12 ± 0.36 to -0.33 ± 0.32 (P < 0.001). Additionally, 43.4% of patients demonstrated significant progress in physical role function, with scores increasing from -2.64 ± 0.44 to -0.99 ± 0.47 (P < 0.001). Regarding psychological health, 36.9% of those initially impaired exhibited substantial improvement, with scores rising from -2.54 ± 0.35 to -0.56 ± 0.38 (P < 0.001). Emotional role function was enhanced in 37.9% of patients, with scores increasing from -3.50 ± 0.48 to -1.35 ± 0.74 (P < 0.001). Social function also showed improvement in 31.0% of patients, with scores rising from -2.97 ± 0.32 to -1.00 ± 0.43 (P < 0.001). The most pronounced improvement was observed in vitality, with 41.9% of patients showing a notable increase in scores from -1.70 ± 0.29 to -0.69 ± 0.44 (P < 0.001). In contrast, patients initially classified as having no significant abnormalities showed minimal changes across most domains. For example, improvements in physical function and overall health were modest (P = 0.065 and P = 0.236, respectively), and there was little to no change in psychological health and emotional role function (P = 0.905). Detailed information on these changes can be found in **Table 2**.

**Table 2:** Post-rehabilitation changes in HRQOL in young and middle-aged patients with first-onset stroke, evaluated by the SF-36 scale.

Function state	Physical functioning				General health			
	n (%)	Pre-rehabilitation	Post-rehabilitation	P value	n (%)	Pre-rehabilitation	Post-rehabilitation	P value
Unremarkable	140 (68.97)	-0.12 ± 0.19	-0.07 ± 0.21	0.065	103 (50.74)	0.09 ± 0.21	0.13 ± 0.18	0.236
Impaired	63 (31.03)	-3.12 ± 0.40	-1.18 ± 0.25	<0.001	100 (49.26)	-2.96 ± 0.47	-1.06 ± 0.79	<0.001

Function state	Physical pain				Physical role function			
	n (%)	Pre-rehabilitation	Post-rehabilitation	P value	n (%)	Pre-rehabilitation	Post-rehabilitation	P value
Unremarkable	150 (73.89)	0.52 ± 0.20	0.52 ± 0.21	0.972	115 (56.65)	-0.79 ± 0.36	-0.20 ± 0.49	<0.001
Impaired	53 (26.11)	-2.12 ± 0.36	-0.33 ± 0.32	<0.001	88 (43.35)	-2.64 ± 0.44	-0.99 ± 0.47	<0.001

Function state	Psychological well-being				Emotional role function			
	n (%)	Pre-rehabilitation	Post-rehabilitation	P value	n (%)	Pre-rehabilitation	Post-rehabilitation	P value
Unremarkable	128 (63.05)	0.11 ± 0.19	0.28 ± 0.30	<0.001	126 (62.07)	0.21 ± 0.11	0.21 ± 0.11	0.905
Impaired	75 (36.95)	-2.54 ± 0.35	-0.56 ± 0.38	<0.001	77 (37.93)	-3.50 ± 0.48	-1.35 ± 0.74	<0.001

Function state	Social functioning				Vitality			
	n (%)	Pre-rehabilitation	Post-rehabilitation	P value	n (%)	Pre-rehabilitation	Post-rehabilitation	P value
Unremarkable	140 (68.97)	0.30 ± 0.24	0.33 ± 0.25	0.18	118 (58.13)	-0.62 ± 0.43	-0.18 ± 0.53	<0.001
Impaired	63 (31.03)	-2.97 ± 0.32	-1.00 ± 0.43	<0.001	85 (41.87)	-1.70 ± 0.29	-0.69 ± 0.44	<0.001

### 3.3 Post-rehabilitation improvements in psychological outcomes

Following rehabilitation training, notable improvements in psychological outcomes were observed in SCL-90 scale. Among patients initially categorized as impaired, 21.2% showed a significant reduction in aggression, with scores decreasing from 67.68 ± 1.78 to 63.93 ± 1.86 (P < 0.001). Similarly, 33.0% of impaired patients exhibited a substantial decrease in anxiety, with scores dropping from 70.80 ± 2.34 to 63.22 ± 2.51 (P < 0.001). Significant improvements were also noted in obsessive-compulsive symptoms and paranoid thoughts. Specifically, 41.9% of impaired patients demonstrated improvements in the obsessive-compulsive dimension, with scores falling from 68.51 ± 2.57 to 62.93 ± 2.52 (P < 0.001). For paranoid thoughts, 11.8% of patients showed a significant reduction in scores, from 63.79 ± 0.87 to 58.74 ± 0.89 (P < 0.001). Insecurity was reduced in 8.9% of patients, with scores decreasing from 69.94 ± 0.94 to 63.74 ± 0.94 (P < 0.001), while somatization symptoms improved significantly in 30.1% of patients, with scores dropping from 64.68 ± 1.80 to 56.00 ± 1.89 (P < 0.001). Psychotic and phobic symptoms also showed noticeable improvements after rehabilitation. Specifically, 35.9% of impaired patients demonstrated improvements in psychotic symptoms, with scores decreasing from 63.82 ± 1.31 to 53.95 ± 1.45 (P < 0.001). Phobic symptoms improved in 25.1% of patients, with scores falling from 65.57 ± 1.86 to 61.95 ± 1.77 (P < 0.001). In contrast, no significant changes were observed in the psychological domains for patients who were initially classified as having no significant abnormalities. Detailed information regarding these changes is available in **Table 3**.

**Table 3:** Post-rehabilitation changes in psychological outcomes in young and middle-aged patients with first-onset stroke, evaluated by the SCL-90 scale.

Function state	Aggressiveness				Anxiety			
	n (%)	Pre-rehabilitation	Post-rehabilitation	P value	n (%)	Pre-rehabilitation	Post-rehabilitation	P value
Unremarkable	160 (78.82)	48.00 ± 2.94	48.06 ± 3.40	0.661	136 (67.00)	46.58 ± 2.48	45.95 ± 2.86	0.063
Impaired	43 (21.18)	67.68 ± 1.78	63.93 ± 1.86	<0.001	67 (33.00)	70.80 ± 2.34	63.22 ± 2.51	<0.001

Function state	Obsessiveness				Paranoid thinking			
	n (%)	Pre-rehabilitation	Post-rehabilitation	P value	n (%)	Pre-rehabilitation	Post-rehabilitation	P value
Unremarkable	118 (58.13)	47.71 ± 2.76	46.91 ± 3.15	0.051	179 (88.18)	43.97 ± 2.59	43.49 ± 2.62	0.078
Impaired	85 (41.87)	68.51 ± 2.57	62.93 ± 2.52	<0.001	24 (11.82)	63.79 ± 0.87	58.74 ± 0.89	<0.001

Function state	Insecurity				Somatization			
	n (%)	Pre-rehabilitation	Post-rehabilitation	P value	n (%)	Pre-rehabilitation	Post-rehabilitation	P value
Unremarkable	185 (91.13)	45.92 ± 2.79	45.49 ± 2.80	0.143	142 (69.95)	47.85 ± 1.87	47.42 ± 1.92	0.051
Impaired	18 (8.87)	69.94 ± 0.94	63.74 ± 0.94	<0.001	61 (30.05)	64.68 ± 1.80	56.00 ± 1.89	<0.001

Function state	Psychoticism				Phobic-anxiety			
	n (%)	Pre-rehabilitation	Post-rehabilitation	P value	n (%)	Pre-rehabilitation	Post-rehabilitation	P value
Unremarkable	130 (64.04)	48.36 ± 1.88	48.36 ± 1.98	0.982	152 (74.88)	48.77 ± 2.80	48.21 ± 2.96	0.095
Impaired	73 (35.96)	63.82 ± 1.31	53.95 ± 1.45	<0.001	51 (25.12)	65.57 ± 1.86	61.95 ± 1.77	<0.001

### 3.4 Post-rehabilitation changes in depression

After rehabilitation, notable reductions in depressive symptoms were observed in young and middle-aged patients with first-onset stroke, as measured by the depressive dimension of the BDI-II and SCL-90 scales. Among the 38.92% of patients initially classified as impaired (n = 79), BDI-II scores decreased substantially from 20.80 ± 1.88 to 11.94 ± 1.88 (P < 0.001). In contrast, among the 61.08% of patients initially classified as having no significant abnormalities (n = 124), changes in depressive scores were relatively modest (P = 0.055). Similarly, significant improvement was noted in the depressive dimension of the SCL-90 scale in the impaired group, with scores dropping from 64.76 ± 1.87 to 58.05 ± 1.86 (P < 0.001). In the group without significant abnormalities, depressive scores showed only a slight decrease, from 13.81 ± 1.85 to 13.35 ± 1.89 (P = 0.056). These results suggest that rehabilitation training had a more pronounced effect in alleviating depression among patients with more severe baseline depressive symptoms, while the improvement in those with milder symptoms was relatively limited. Further details can be found in **Table 4**.

**Table 4:** Post-rehabilitation changes in depressive symptoms in young and middle-aged patients with first-onset stroke, evaluated by the BDI-II and SCL-90 scales.

Function state	BDI-II				SCL-90			
	n (%)	Pre-rehabilitation	Post-rehabilitation	P value	n (%)	Pre-rehabilitation	Post-rehabilitation	P value
Unremarkable	124 (61.08)	5.42 ± 0.93	5.20 ± 0.91	0.055	118 (58.13)	13.81 ± 1.85	13.35 ± 1.89	0.056
Impaired	79 (38.92)	20.80 ± 1.88	11.94 ± 1.88	<0.001	85 (41.87)	64.76 ± 1.87	58.05 ± 1.86	<0.001

## 4 Discussion

This study seeks to explore the effects of structured rehabilitation programs on psychological outcomes and HRQOL in young and middle-aged individuals with first-onset stroke. The findings suggest that comprehensive rehabilitation, which includes physical therapy, occupational therapy, and psychological counseling, offers significant improvements in both psychological well-being and HRQOL, especially for those with more severe impairments prior to rehabilitation. These results underscore the value of an integrated rehabilitation approach that not only focuses on physical recovery but also emphasizes psychological recovery. The study is particularly noteworthy for its focus on the often underrepresented group of young and middle-aged stroke patients in rehabilitation research, highlighting the positive impact of holistic rehabilitation strategies on improving both their psychological health and quality of life.

This study observed improvements in HRQOL, especially in the “impaired” group, as measured by the SF-36 scale. After rehabilitation, patients showed noticeable gains in physical function, overall health, bodily pain, and vitality. These findings align with previous research, which has also suggested that stroke patients tend to recover some aspects of physical function following rehabilitation (24, 25). Stroke rehabilitation is a multifaceted process designed to restore functional abilities and enhance overall quality of life (26–29). It involves a combination of therapeutic interventions, including physical, occupational, and psychological therapies, that target both the physical impairments and the psychological and emotional challenges resulting from stroke (26–29). A key goal of rehabilitation is to enhance HRQOL, which encompasses an individual’s overall well-being in multiple areas, such as physical, psychological, and social functioning (30, 31). HRQOL is a multifaceted concept that aims to capture how an individual’s health condition influences different areas of daily living (32, 33). For stroke survivors, HRQOL includes aspects such as physical functioning, pain, social interaction, psychological well-being, and independence in daily activities (34, 35). Improvements in HRQOL following stroke rehabilitation have been consistently observed across various studies. For instance, research by Gao et al. (36) suggested that non-pharmacological interventions, including exercise training, constraint-induced movement therapy, and music therapy, were associated with improvements in various aspects of HRQOL in stroke survivors, affecting physical, psychological, and social dimensions. Similarly, Visser et al. (37) found that integrating problem-solving therapy into stroke outpatient rehabilitation led to improvements in task-oriented coping strategies and overall HRQOL. However, this intervention did not show a significant effect on psychosocial quality of life specific to stroke. In a separate study, Brouns et al. (38) observed that eRehabilitation, which integrates cognitive and physical training, activity monitoring, and psychological education, resulted in noticeable improvements in communication abilities and physical strength when combined with traditional rehabilitation methods. While the improvements were relatively modest, this approach appeared to support the long-term enhancement of HRQOL. Together, these studies highlight the diverse approaches in stroke rehabilitation that can contribute to improvements in different aspects of HRQOL. Psychological rehabilitation is an important factor in enhancing HRQOL after a stroke. Stroke survivors frequently face challenges such as depression, anxiety, and cognitive impairments, which can considerably affect their overall HRQOL (39, 40). Psychological counseling and cognitive-behavioral therapies have been shown to reduce depression and anxiety while enhancing emotional regulation and coping skills, thus contributing to improved psychological well-being. For instance, Zhao et al. (41) found that psychological interventions, when incorporated into rehabilitation therapy, can assist stroke patients with anxiety and depression in improving their ability to carry out daily tasks, enhancing overall quality of life, and alleviating symptoms of both anxiety and depression. Additionally, rehabilitation’s impact on HRQOL can vary depending on the stroke severity and the timing of rehabilitation. Early intervention tends to yield more favorable outcomes, as patients can regain more function when rehabilitation is initiated sooner (42, 43). However, even patients with more severe impairments can show significant improvements in HRQOL with long-term rehabilitation, although these improvements may be more gradual and less pronounced compared to those with mild impairments. In this study, the “Unremarkable” group showed minimal improvements, suggesting that patients with milder impairments may benefit less from rehabilitation, possibly due to a ceiling effect in domains like physical functioning and general health. This highlights the need for creating personalized rehabilitation plans that cater to the physical and psychological requirements of stroke patients.

Furthermore, after rehabilitation, significant improvements were observed in both psychological outcomes and depressive symptoms, particularly in the “Impaired” group. Psychological outcomes, as assessed by the SCL-90 scale, showed notable reductions in aggressiveness, anxiety, obsessiveness, and paranoid thinking, reflecting the effectiveness of the psychological counseling component of the rehabilitation program. Additionally, following the evaluation with the BDI-II and SCL-90 depression scales, a notable decrease in depressive symptoms was observed in the “impaired” group. The minimal changes observed in the “Unremarkable” group suggest that patients with milder depressive symptoms may not benefit as much from psychological interventions, reinforcing the need for early and comprehensive interventions in patients with more severe psychological impairments. Stroke frequently results in a range of psychological challenges, including depression, anxiety, and mood fluctuations, all of which can adversely affect the rehabilitation process and overall quality of life for patients (41, 44–46). Zheng et al. (40) found that depression and cognitive impairment are relatively common among elderly stroke survivors in China, with a notable relationship between the two conditions. The severity of these symptoms appears to be linked to a considerable decline in the patients’ quality of life. Psychological distress in stroke survivors not only hinders rehabilitation progress but also exacerbates physical impairments, leading to worse long-term outcomes (12, 47, 48). Rehabilitation programs that integrate psychological counseling, cognitive behavioral therapy, and psychoeducation have shown to effectively alleviate these psychological symptoms, thus enhancing both emotional well-being and physical recovery. Multiple studies have shown that rehabilitation can help reduce symptoms of depression and anxiety following a stroke, underscoring the value of using a comprehensive, multidisciplinary approach to manage both the physical and psychological challenges faced by patients (49–51). Lei et al.’s review (52) suggested that psychological interventions, including cognitive-behavioral therapy and supportive psychotherapy, may contribute to improvements in the psychological well-being, cognitive function, and physical recovery of stroke patients dealing with anxiety and depression. Similarly, Nungulo et al. (53) found that psychological education interventions can contribute to improvements in self-care abilities, functional independence, and the ability to manage depression and anxiety in stroke patients, ultimately supporting overall health and quality of life. Psychological challenges are especially notable in younger stroke patients. Young stroke survivors face unique psychological experiences and challenges, including premature disability, emotional adjustment, and the impact of their condition on family and social roles (54, 55). In their meta-analysis on post-traumatic growth in young and middle-aged stroke patients, Liu et al. (56) outlined a three-phase framework, which includes trauma and stress, adaptation and reconstruction, and growth. This framework underscores the importance of recovery across physical, psychological, and social domains throughout the rehabilitation process. The psychological symptoms in this group, including anxiety, depression, and post-traumatic stress, can be more pronounced due to their unexpected and life-altering nature (57, 58). This younger demographic often struggles with issues such as career disruption, reduced social engagement, and changes in family dynamics, further exacerbating their mental health concerns (57, 58). The urgency of addressing these issues is heightened by the long-term nature of psychological recovery and the significant impact it has on functional outcomes. Addressing the psychological needs of young stroke survivors through effective rehabilitation is essential. This approach not only supports emotional recovery but also aids in their successful reintegration into society. This study is valuable in contributing to the existing literature by focusing on the psychological outcomes of young and middle-aged stroke survivors, a group that is often underrepresented in current rehabilitation research. The findings highlight the effectiveness of a comprehensive rehabilitation approach in improving psychological well-being and reducing depressive symptoms, especially for those with more severe impairments.

Interestingly, the study observed that the “impaired” group showed greater improvements in psychological outcomes and HRQOL following rehabilitation compared to the “non-impaired” group. Several interrelated mechanisms may explain this difference. First, individuals with greater baseline deficits may have more “room for improvement,” as they have a larger margin for measurable change (59, 60). This could result in more substantial absolute improvements in the impaired group. Second, neuroplastic and behavioral adaptation processes may be more readily triggered when impairments are more severe. The higher intensity of rehabilitation demands may stimulate greater cognitive and emotional re-engagement, motivation, and therapeutic responsiveness. Previous research suggests that individuals with functional impairments are more vulnerable to psychological distress, which may drive stronger motivation for rehabilitation in the impaired group (61–63). Lastly, the non-impaired group may already function at or near normative levels in many domains at baseline, which limits the potential for change during the short rehabilitation period, thus reducing the measurable improvements achievable. These findings suggest that a one-size-fits-all approach to rehabilitation may not be optimal for the non-impaired group. For these individuals, the focus could shift from remediation to enhancement, maintenance, and participation-oriented interventions. Strategies might include strengthening resilience, promoting social reintegration, and providing preventive psychological education. The criteria for tailoring rehabilitation could involve baseline psychological scores, the presence of sub-clinical symptoms, and functional or social role participation status (64, 65). For example, non-impaired participants with high baseline scores but concerns about social reintegration might benefit more from group-based psychosocial programs or goal-setting interventions rather than the standard therapies applied to impaired individuals. Future studies should stratify participants by impairment status and potential for change, testing adaptive rehabilitation approaches tailored to individual needs. Longitudinal follow-up is needed to determine if the smaller improvements in the non-impaired group reflect a true plateau or require longer or different interventions. Further research on motivational, psychological, and neurobiological factors in non-impaired stroke survivors could help optimize rehabilitation strategies for this subgroup.

Comorbidities such as diabetes mellitus, hypertension, hyperlipidemia, and coronary artery disease can significantly influence both psychological functioning and HRQOL in stroke patients. These conditions often exacerbate physical disability, increase psychological distress, and reduce the overall effectiveness of rehabilitation programs (66, 67). Previous studies have shown that individuals with comorbidities tend to experience more severe psychological symptoms, such as depression and anxiety, which can impede recovery and worsen HRQOL outcomes (68–70). In particular, conditions like diabetes and hypertension are associated with increased levels of stress, reduced mobility, and poorer physical functioning, all of which can negatively impact rehabilitation outcomes (71, 72). Additionally, comorbidities often contribute to a higher burden of symptoms, which may limit the capacity for meaningful improvement in both psychological well-being and physical function. The cumulative effect of multiple comorbid conditions can result in a more complex rehabilitation process, requiring tailored interventions that address both the physical and psychological challenges these patients face. Future research should explore how comorbidities affect rehabilitation effectiveness and recovery, focusing on their impact on psychological well-being and HRQOL. This understanding can help develop more effective, individualized rehabilitation strategies for these patients.

While this study offers important insights into the effects of rehabilitation on psychological outcomes and HRQOL, there are some limitations. First, the study relied on self-reported psychological outcomes, which may introduce potential biases. The use of more objective methods, such as clinical evaluations or neuroimaging data, could provide a more thorough assessment of psychological and neurobiological changes following rehabilitation. Second, the study focused primarily on a homogeneous group of participants, limiting the generalizability of the findings to more diverse populations with varying types and severities of stroke. Finally, future studies should examine the long-term impact of rehabilitation on psychological health and HRQOL, as the current research focused solely on short-term outcomes.

## 5 Conclusion

In summary, this study demonstrates that rehabilitation is associated with meaningful improvements in psychological outcomes and HRQOL among young and middle-aged stroke patients, with the greatest benefits observed in those who had more severe impairments before starting rehabilitation. The findings stress the importance of combining both physical and psychological interventions in post-stroke rehabilitation to promote comprehensive recovery. Early implementation of tailored psychological rehabilitation strategies can not only enhance emotional adjustment but also support functional recovery and lead to broader improvements in quality of life for stroke survivors.

## Data Availability

The raw data supporting the conclusions of this article will be made available by the authors, without undue reservation.
